# Radiation protection for comforters and carers in radiology and nuclear medicine

**DOI:** 10.1002/jmrs.683

**Published:** 2023-04-24

**Authors:** Mohamed K. Badawy, Alice Anderson

**Affiliations:** ^1^ Monash Health Imaging Monash Health Clayton Victoria Australia; ^2^ Department of Medical Imaging and Radiation Sciences, School of Primary and Allied Health Care, Faculty of Medicine, Nursing and Health Sciences Monash University Clayton Victoria Australia; ^3^ Monash Health Library Services Monash Health Clayton Victoria Australia

Radiation exposure concerns all individuals in radiology and nuclear medicine (NM), including comforters and carers (referred to as ‘carers’ henceforth). Carers play a critical role in the medical radiation exposure setting, providing essential support and comfort to patients undergoing radiological or NM procedures for medical diagnosis or treatment. In this context, carers provide this care voluntarily and separately from their occupation. It is essential to take precautions to protect their health.

Carers may be present during radiological procedures, such as X‐rays and CTs, or NM procedures, such as radiopharmaceutical administration, imaging or care for therapy patients. These individuals are often close to radiation sources, the primary beam or the patient. They may be at risk for radiation‐induced side effects if appropriate precautions are not taken. These precautions may include using protective equipment, for example, radioprotective aprons or shields, increasing distance from the source and minimising time spent near the radiation source.

## Radiation risks to the carer

Radiation exposure and its potential health effects are well documented. However, for carers, the exposure is typically below 1 mSv, which does not pose a significant risk of radiation‐induced carcinogenesis or tissue reactions. Although the literature does not provide clear evidence for an increased risk of cancer or tissue reactions at this dose range, the assumption of a linear no‐threshold model for radiation risk suggests that any increase in exposure could potentially lead to an increased risk of future cancers. It is important to consider carers' age in radiation risk communication, as it may impact their understanding and perception of risk. To help carers better understand the risk, it can be helpful to compare the radiation exposure from medical procedures to everyday activities, such as air travel.

The potential psychological effects of radiation exposure on carers have yet to be documented in the literature. Nevertheless, it is important to recognise that anxiety and concerns about possible health risks associated with radiation exposure can be a genuine issue for this population. To support carers and address their anxieties, it is crucial to provide them with information about the risks of radiation exposure and an opportunity to ask questions during the informed consent process. Additionally, providing shielding in low‐exposure settings, even if it does not directly reduce radiation risk, can help alleviate some anxieties. However, shielding should not be used as a substitute for education.

## Practice guidance and legal requirements

The International Commission on Radiological Protection (ICRP) recommends that carers have a dose constraint of 5 mSv per episode of care.^1^ In Australia, the Code for Radiation Protection in Medical Exposure^2^ guides the protection of these carers by setting two different dose constraints. During radiological examinations, the dose constraint is set at 1 mSv, the same as the public. However, for treatment settings, such as radionuclide therapy, the dose constraint is set at a higher level of 5 mSv. It's important to note that dose constraints, unlike regulatory dose limits, serve as upper limits to prospectively guide individuals in reducing radiation exposure to as low as reasonably achievable levels, bearing in mind economic and social factors. While dose constraints guide minimising exposure, they can be used flexibly in exceptional circumstances, such as caring for very sick children, where higher carer doses may be necessary.^1^

Informed consent is crucial for carers. It ensures that these individuals are aware of the potential risks of exposure to ionising radiation and can make informed decisions about their involvement. Given that carers may receive higher doses of radiation than the public, it is essential that they fully understand the risks and benefits of their involvement. Their informed consent should be documented in the hospital's record‐keeping system. However, obtaining informed consent from carers can pose logistical challenges, and hospitals must consider these implications in their duty of care.

## Radiology

The studies by Kostidis et al.,^3^ Perdomo et al.^4^ and Suleiman et al.^5^ provide valuable information about the magnitude of radiation exposure for carers in mammography and paediatric X‐ray exams. These studies demonstrate low exposure and contribute important data for informed consent and local procedure development. Notably, the recent studies by Kostidis et al. and Perdomo et al. offer insights while considering more recent technology, practice and attitudes towards radiation protection for comforters and carers. Due to the paucity of existing evidence, these studies are particularly significant.

However, it is important to note that these studies cannot be generalised to other situations or modalities, such as CT and Fluoroscopy. A recent study on paediatric CT scans found that badges worn by carers during real exams have never recorded a dose above the minimum reportable level (1 μSv).^6^ The maximum dose received from ten consecutive simulated scans was only 3 μSv. Further research is needed to fully comprehend radiation exposure in radiology, particularly in high‐exposure settings like CT. Since each care situation is unique, future studies should focus on maximum likely exposure.

## Nuclear medicine exposure situations

Diagnostic NM procedures can potentially produce higher radiation exposure than radiology. Despite the limited recent research on the topic, a study by Díaz Barreto et al.^7^ investigated two scenarios where a carer's exposure could be high and found that the dose was unlikely to exceed 1 mSv.

A comprehensive examination of the literature published before 2014 on radionuclide therapy was conducted by Stefanoyiannis et al.^8^ The findings suggest that carers will likely receive less than 1 mSv of exposure while caring for patients following therapy. This conclusion is supported by a study by Gains et al.^9^; however, they reported a rare instance when caring for a patient administered with I‐131, where the carer's dose exceeded 5 mSv due to the patient being a 2‐year‐old with visual impairment and wearing diapers, thus requiring extra care.

Regulations require patients undergoing therapeutic procedures with high doses of radiation to be isolated in the hospital until they are no longer a risk to others. As a result, it is unusual for carers to exceed this level unless exceptional circumstances arise, as seen in the case described by Gains et al.^9^ However, in NM, the safety precautions and recommendations can be tailored to each case by measuring the dose rate at a distance from the patient and estimating the worst‐case scenario for the carer. Recommendations should emphasise increasing the distance from the patient when possible and reducing time spent in close proximity to minimise the carer's exposure.

## Protection

The exposure level can be reduced by applying the principles of time, distance and shielding. For instance, increasing distance from the radiation source and reducing exposure time can help lower the dose carers receive. This can be achieved by limiting the number of views the carer attends or standing in a position that increases the distance from the source. Considering the linear no‐threshold assumption, shielding with radioprotective personal protective equipment (PPE) or fixed or mobile shields can lower the dose and potentially impact risk reduction; however, the precise extent of this impact remains unclear at a very low radiation dose. For example, data from studies such as Perdomo et al.^4^ and Kostidis et al.^3^ indicate that without shielding, carers would receive less than 2 μSv, equivalent to a few hours of naturally occurring background radiation. This would not pose a significant risk. However, shielding may provide psychological benefits for carers, such as reducing anxiety. In CT and Fluoroscopy, guidance can be obtained from vendor‐provided isodose curves or local measurements of scatter using phantoms. In most cases, using radioprotective shielding for these modalities will be appropriate and provide some benefit.

In NM, the use of shielding may reduce radiation exposure. However, it is essential to note that the shielding efficiency decreases as the radionuclide energy increases, and in some cases, the dose may even increase.^10^ Additionally, the longer exposure from a patient in NM may make it impractical to use radioprotective PPE, which can cause other issues, such as back problems. For therapeutic procedures, shielding can become completely impractical when caring for a patient outside of hospital and using time and distance will be more beneficial.

In all settings, advice should be evaluated on a case‐by‐case basis. A standard approach to shielding should be established to ensure clarity among carers who may accompany patients to different medical institutions. Without college or regulatory guidance, each site will develop its procedures, which may differ. For example, Perdomo et al.^4^ proposed that only carers likely receiving more than 12 hours of background radiation would be given PPE. In contrast, at the Authors' institute, all carers are given aprons in radiology and consideration is given in NM imaging on a case‐by‐case basis.

## Data Availability

Data sharing not applicable to this article as no datasets were generated or analysed during the current study.

## References

[1] International Commission on Radiological Protection . ICRP Publication 103 [Internet]. 2007. Available from: https://www.icrp.org/publication.asp?id=ICRP%20Publication%20103 10.1016/j.icrp.2007.10.003 18082557

[2] Australian Radiation Protection and Nuclear Safety Agency . Code for Radiation Protection in Medical Exposure, Radiation Protection Series C‐5 [Internet]. 2019. Available from: https://www.arpansa.gov.au/sites/default/files/medical‐exposure‐code‐rps‐c‐5.pdf

[3] Kostidis M , Varcoe J , Barnes P . Assessment of scatter radiation dose received by comforters and carers during digital breast tomosynthesis mammography. J Med Radiat 2023; 70: 112–9. 10.1002/jmrs.649 PMC10258642 36852488

[4] Perdomo A , McMahon S , Wilkie T , Fox N , Rao P . Do carers and comforters require lead aprons during general radiographic examinations? J Med Imaging Radiat Oncol 2022; 66: 25–33. 34331404 10.1111/1754-9485.13304

[5] Sulieman A , Vlychou M , Tsougos I , Theodorou K . Radiation doses to paediatric patients and comforters undergoing chest X rays. Radiat Prot Dosimetry 2011; 147: 171–5. 21743069 10.1093/rpd/ncr295

[6] Overhoff D , Weis M , Riffel P , et al. Radiation dose of chaperones during common pediatric computed tomography examinations. Pediatr Radiol 2020; 50: 1078–82. 32415324 10.1007/s00247-020-04681-6 PMC7329757

[7] Díaz Barreto M , López Bejerano GM , Varela Corona C , Fleitas EI . On the safety of persons accompanying nuclear medicine patients. Radiat Prot Dosimetry 2012; 152: 313–6. 22517979 10.1093/rpd/ncs055

[8] Stefanoyiannis AP , Ioannidou SP , Round WH , et al. Radiation exposure to caregivers from patients undergoing common radionuclide therapies: a review. Radiat Prot Dosimetry 2015; 167: 542–51. 25431487 10.1093/rpd/ncu338

[9] Gains JE , Walker C , Sullivan TM , et al. Radiation exposure to comforters and carers during paediatric molecular radiotherapy. Pediatr Blood Cancer 2015; 62: 235–9. 25284346 10.1002/pbc.25250

[10] Deb P , Jamison R , Mong L , Ujj P . An evaluation of the shielding effectiveness of lead aprons used in clinics for protection against ionising radiation from novel radioisotopes. Radiat Prot Dosimetry 2015; 165: 443–7. 25848112 10.1093/rpd/ncv065

